# Benefits of Local Treatment Including External Radiotherapy for Hepatocellular Carcinoma with Portal Invasion

**DOI:** 10.3390/biology10040326

**Published:** 2021-04-14

**Authors:** Han Ah Lee, Sunmin Park, Yeon Seok Seo, Won Sup Yoon, Chai Hong Rim, on behalf of the Korean Liver Cancer Study Group

**Affiliations:** 1 Department of Internal Medicine, Sanggye Paik Hospital, Inje University College of Medicine, Seoul 01757, Korea; amelia86@korea.ac.kr; 2 Department of Internal Medicine, Korea University Anam Hospital, Korea University Medical College, Seoul 02841, Korea; gandorie@gmail.com; 3 Department of Radiation Oncology, Korea University Ansan Hospital, Korea University Medical College, Gyeonggido 15355, Korea; sunmini815@kumc.or.kr (S.P.); irionyws@korea.ac.kr (W.S.Y.)

**Keywords:** radiotherapy, portal vein thrombosis, BCLC C, ALBI grade

## Abstract

**Simple Summary:**

The benefit of local treatment for hepatocellular carcinoma (HCC) with portal invasion is unclear. Radiotherapy can technically palliate vessel-invasive HCC; however, the survival benefit has not been confirmed. Local treatment including radiotherapy showed a survival benefit in large propensity score-matched cohorts (median survival: 8 vs. 2 months, *p* < 0.001). The benefit persisted among patients with Child-Pugh class A and B liver function. Our results represent community-level data from all Korean administrative districts.

**Abstract:**

We aimed to identify the oncologic benefits of local treatment including radiotherapy (LRT) in hepatocellular carcinoma (HCC) invading the portal vein. We used clinical data of patients with HCC invading the portal vein from 2008 to 2014 provided by 50 hospitals nationwide. A total of 1163 patients were included in the analysis. The LRT group was younger than the best supportive care (BSC) group (*p* < 0.001). The mean Child-Pugh score of the LRT group (6.1) was significantly lower than that of the BSC group (7.7) (*p* < 0.001). Propensity score-matched analysis generated 222 pairs. The median survival of all patients, LRT, and BSC groups were 5.0, 8.0, and 2.0 months, respectively. The overall survival (OS) rates in the LRT and BSC groups were 34.2% and 16.2% at one year, and 12.6% and 6.8% at two years, respectively (*p* < 0.001). Multivariate analysis showed that LRT (HR 0.41, 95% CI 0.32–0.52), age >60 years, extrahepatic metastases, tumor size ≥10 cm, and Child-Pugh class (CPC) B or C were independent predictors of higher mortality (all *p* < 0.05). Statistical differences in survival were maintained in all CPC-albumin-bilirubin classes (all *p* < 0.05). LRT was significant in patients with HCC with portal invasion, valid for patients with CPC A and B.

## 1. Introduction

Hepatocellular carcinoma (HCC) is prevalent in East Asia, and its most common cause is hepatitis B virus (HBV) mutagenesis [1]. Due to the characteristics of HBV-related HCC, which tends to progress locally rapidly and invades major vessels, it is often unresectable at diagnosis [2]. In unresectable HCCs, transarterial chemoembolization (TACE) or systemic therapy is commonly performed [3,4]. Although TACE is an efficient treatment, obtaining a complete response in large or vessel-invasive tumors is difficult [5]. Sorafenib has been solely recommended by most guidelines, showing extended overall survival (OS); however, its response rate is unsatisfactory (<3%) [6,7]. Furthermore, it might be difficult to apply in patients with impaired liver function or in developing countries that lack established financial support [8,9,10].

Portal invasion is one of the most common causes of unresectable HCC [11]. External-beam radiation therapy (EBRT) has the advantage of being applied regardless of the tumor location or vessel involvement [12]. Therefore, EBRT has been commonly used as a palliative modality for locally advanced tumors, particularly in East Asian countries [13,14]. In a surveillance study of 161 HCC clinicians in Korea, 70–85% of doctors responded that they would prescribe EBRT for HCC with major vessel invasion or with an incomplete response after TACE [15]. However, most studies reporting the effectiveness of EBRT for HCC are case series [14], and Phase 3 studies demonstrating clear survival benefits are yet to be published.

In real-world practice, for unresectable cases including those with major vessel invasion, many community hospitals do not attempt active oncologic therapy. In our previous study regarding data from nationwide hospitals in South Korea, more than half of the HCC patients with portal invasion received supportive care without active oncologic treatment [11]. In Southeast Asian countries such as Vietnam and Cambodia, the vast majority of patients with HCC receive palliative care alone [8,10]. This phenomenon is possibly due to economic reasons as well as because some community physicians believe that active local treatment has unclear benefits for unresectable cases.

Randomized studies to evaluate the benefits of implementing local treatment including radiotherapy (LRT) might be difficult to design due to the expected hazard of the group who did not receive LRT. Therefore, in this study, the oncologic benefit of LRT was evaluated using data randomly extracted from over 50 hospitals in all administrative districts in Korea with the help of propensity-score (PS) matching.

## 2. Materials and Methods

### 2.1. Data Source

The Ministry of Health and Welfare of Korea initiated the Korean Central Cancer Registration (KCCR) project in 1980. The Korean Liver Cancer Study Group (KLSCG) extracted data from patients with HCC using the ICD-10 code C22.0 from the KCCR database. The KLSCG implemented three projects: 2008–2010, 2011–2012, and 2013–2014, and each project included 47, 50, and 51 hospitals, respectively. Each project included a minimum of one of the 16 major administrative districts in Korea. In the selection of hospitals, a probability proportional extraction method was used to ensure that hospitals with more patients with HCC were more likely to be selected. Selected hospitals randomly extracted patients diagnosed with HCC and provided clinical data of approximately 16.5% of all patients. Through three projects, data on 10,580 patients diagnosed with HCC from 2008 to 2014, corresponding to 11.7% of all HCC patients nationwide, were obtained.

### 2.2. Study Cohort and Propensity-Score Matching

The database contains information on the first and second treatments. In this study, initial treatments were defined to include the first and second treatments, with the latter applied within two months after the first treatment. The target cohort of this study included patients with HCC and portal vein invasion. The intervention arm of this study was defined as patients who underwent LRT as the initial treatment. The control group in this study was the best supportive care (BSC) group, which was defined as receiving no oncologic treatment at the initial clinical decision.

### 2.3. CPC-ALBI Class

Since approximately two-thirds of patients had liver function of Child-Pugh class (CPC) A, and the benefit of LRT might be hindered by radiation-induced hepatic toxicity, we further utilized the albumin-bilirubin (ALBI) grade [16] to create subdivided stages. Patients with CPC A and ALBI grades 1 and 2 were classified as CPC-ALBI class A1 and A2, respectively, and patients with Child-Pugh class B or higher were classified as CPC-ALBI class B or higher. Using the CPC-ALBI class, we attempted to analyze whether there was a difference in the LRT benefit according to the degree of reserved liver function.

### 2.4. Statistical Analyses

The primary endpoint of this study was OS, and the secondary endpoint was the cause-specific survival (CSS). The Kaplan–Meier estimate [17] was used for survival analysis, and the log-rank test was performed for univariate intergroup comparisons. A Cox proportional hazards model was used for multivariate analysis [18]. To reduce selection bias and the effect of potential confounders, PSs were calculated using logistic regression based on age, Child-Pugh score, etiology of underlying liver disease, presence of extrahepatic metastases (EHM), and tumor number. The chi-squared test was performed for comparison between propensity-matched groups. Differences between the two groups were balanced by 1:1 PS-matched analysis with the nearest-neighbor method, with a minimum *p*-value of 0.2, for all included variables. All statistical analyses were performed using Web-based Analysis (R 4.0; https://cardiomoon.shinyapps.io/webr/, accessed on 1 December 2020).

### 2.5. Ethical Approval and Informed Consent

Our study was evaluated and approved by the Institutional Review Board of Korea University Medical Center (IRB number: 2021AS0034), and informed consent was waived because our study did not pose any harm to the patients involved and no personally identifiable information was used.

## 3. Results

### 3.1. Descriptive Analysis

**Figure 1:**
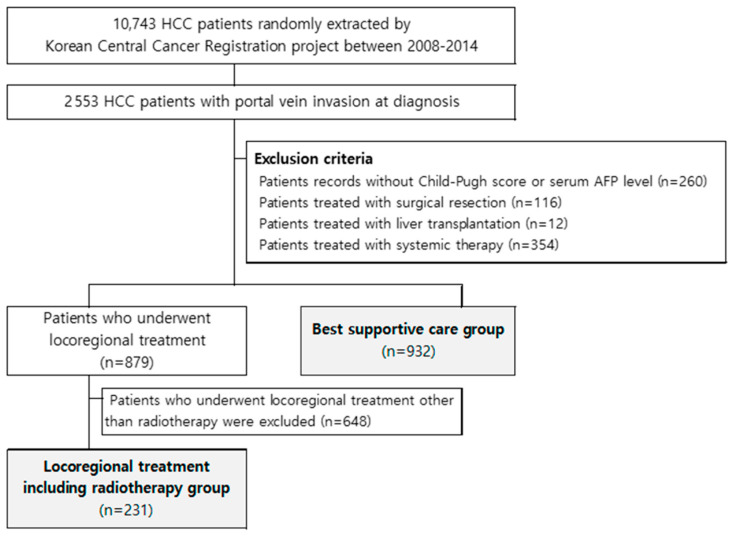
Patient selection process.

Between 2008 and 2014, 10,743 patients with HCC were sampled using random extraction methods, and approximately one-quarter (23.8%, 2553 patients) were found to have portal vein invasion at diagnosis. The exclusion criteria were as follows: (1) patient records with missing values in Child-Pugh score or serum alpha-fetoprotein (AFP) level; (2) patients treated with surgical resection; (3) those treated with liver transplantation; and (4) those treated using systemic therapy. Thereafter, 879 patients who underwent local treatment and 932 with BSC remained. After excluding 648 patients who underwent local treatment other than radiotherapy, 231 patients treated with LRT were finally selected for analysis (Figure 1).

### 3.2. Baseline Characteristics

A total of 1163 patients were analyzed, and their baseline characteristics are presented in Table 1. The LRT group was significantly younger than the BSC group (54.6 years vs. 60.4 years, *p* < 0.001). The mean Child-Pugh score of the LRT group was 6.1, which was significantly lower than that of the BSC group (7.7) (*p* < 0.001). Patients with HBV infection were more prevalent in the LRT group than in the BSC group (77.6% vs. 64.3%, *p* < 0.001), and 51.9% of patients in the LRT group had a single HCC, which was significantly different from that of the 39.3% of patients in the BSC group (*p* = 0.001). Extrahepatic metastases were less prevalent in the LRT group than in the BSC group (23.45 vs. 31.3%, *p* = 0.023).

### 3.3. Propensity-Score Matching

The 1:1 PS-matched analysis generated 222 pairs, and detailed information of the matched cohorts is shown in Table 1. The mean age, Child-Pugh score, prevalence of HBV infection and extrahepatic metastases, and proportion of patients with single tumors were comparable between the two groups (all *p* > 0.2, except age [*p* = 0.165]).

### 3.4. Analyses of Survival Endpoints

A total of 444 patients, matched in two groups (222 patients each in the LRT and BSC groups), were included in the endpoint analyses. During a median follow-up of 5.0 months (range: 1–117 months), 422 patients (99.8%) in the LRT group died, and 409 patients (92.1%) in the BSC group died. The median survival of all patients in the LRT and BSC groups was 5.0 (95% CI, 4.6–5.3), 8.0 (95% CI 7.0–8.9), and 2.0 (95% CI, 1.8–2.3 months), respectively. The OS rates in the LRT and BSC groups were 34.2% and 16.2% at one year, and 12.6% and 6.8% at two years, respectively (*p* < 0.001).

**Figure 2:**
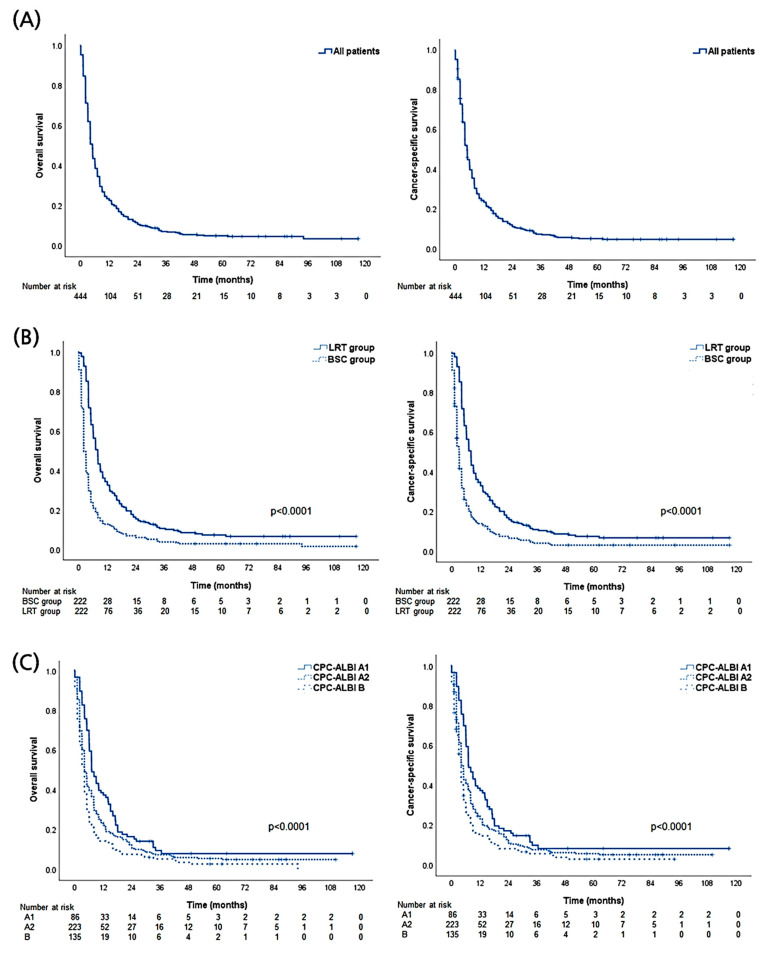
Descriptive graphs of overall and cause-specific survival. (**A**) All propensity-score matched patients. (**B**) Local treatment including radiotherapy (LRT) versus best supportive care (BSC) groups. (**C**) Comparison among subgroups according to Child-Pugh class (CPC) and Albumin-Bilirubin index (ALBI).

In univariate analyses, LRT was significantly associated with a higher OS rate (*p* < 0.001). Age >60 years, presence of EHM, main tumor size ≥10 cm, multiple tumors, higher AFP level, CPC B or C, and performance status ≥2 were significantly associated with higher mortality (all *p* < 0.05). Further multivariate analysis showed that LRT (HR 0.41; 95% CI, 0.32–0.52; *p* < 0.001) was an independent predictor of lower mortality. Additionally, age >60 years (*p* < 0.001), presence of EHM (*p* < 0.001), main tumor size ≥10 cm (*p* < 0.001), and CPC B or C (B vs. A, *p* < 0.001; C vs. A, *p =* 0.016) were independent predictors of higher mortality in patients with HCC invading the portal vein. The results of analyses regarding CSS were mostly similar to those regarding OS (>95% of overall death cases were caused by HCC) (Table 2). Descriptive OS and CSS graphs of all patients, those categorized with initial treatments (LRT vs. BSC), and CPC-ALBI class are shown in Figure 2.

### 3.5. The Benefit of Survival According to CPC-ALBI Class

**Figure 3:**
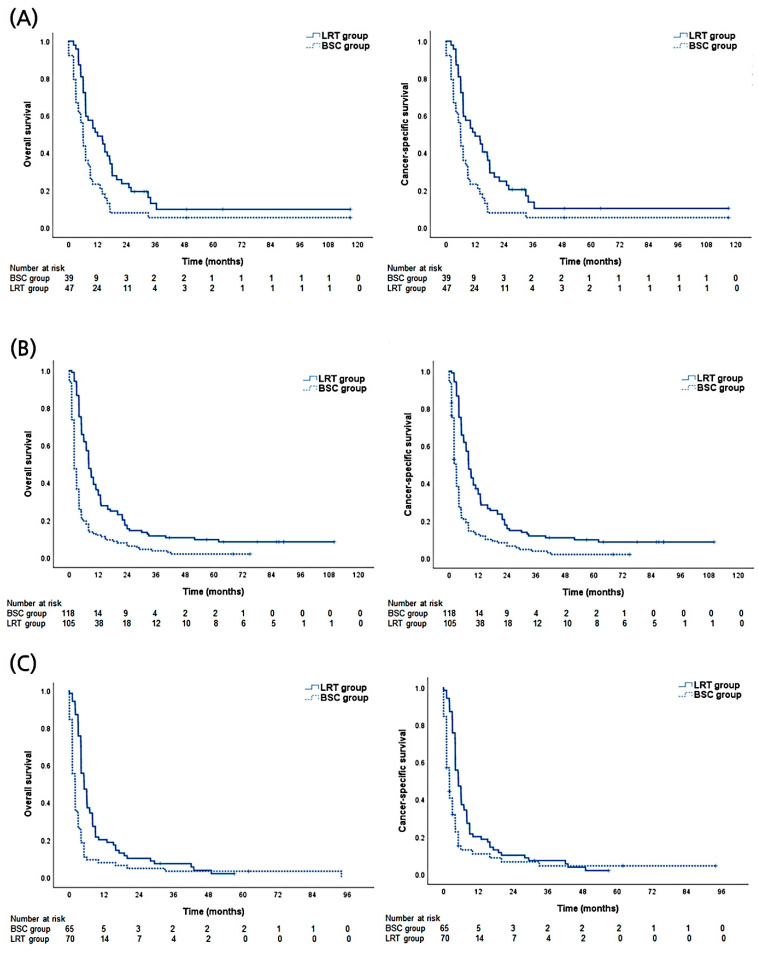
Descriptive graphs of overall and cause-specific survival according to treatment modalities (local treatment including radiotherapy [LRT] versus best supportive care [BSC] groups) of (**A**) Child-Pugh class (CPC) and albumin-bilirubin index (ALBI) A1 group. (**B**) CPC-ALBI A2 group. (**C**) CPC-ALBI B or higher group.

The survival benefit of LRT was further analyzed according to the CPC-ALBI class (Figure 3). In the CPC-ALBI A1 group, the 1- and 2-year OS rates of the LRT arm were 51.1% and 23.4%, respectively, while the corresponding rates of the BSC arm were 23.1% and 7.7%, respectively (*p* = 0.003). Statistical differences in survival were also observed in CPC-ALBI A2 (1- and 2-year OS rates of 36.2% and 17.1% in the LRT arm vs. 11.9% and 7.6% in the BSC arm, *p* < 0.001) and B or higher groups (1- and 2-year OS rates of 20.0% and 10.0% in the LRT arm vs. 7.7% and 4.6% in the BSC arm, *p* < 0.001).

In the LRT group, TACE was combined as an initial treatment in 144 patients (64.9%). Subgroup analyses were performed to investigate the treatment effect of TACE as a combination therapy in the LRT group. Clinical characteristics were comparable between those receiving LRT alone and those receiving LRT with TACE (all *p* > 0.05) (Supplementary Table S1). There was a non-significant trend of higher OS in those receiving LRT with TACE than in those receiving LRT alone (*p* = 0.089). In Cox regression analysis, the combination of TACE as the initial treatment was not significantly associated with OS (HR 0.78; 95% CI, 0.59–1.04; *p* = 0.093) or CSS (HR 0.79; 95% CI, 0.59–1.05; *p* = 0.105).

## 4. Discussion

Our study successfully demonstrated the benefit of LRT for HCC with portal invasion, in terms of OS and CSS, when compared with BSC. Both endpoints were significantly higher with the application of LRT in univariate analyses after PS matching and multivariate analyses. The benefit of LRT persisted regardless of liver function, from CPC A to B, and in subgroups categorized combined with the ALBI score. Furthermore, our study has the merit of demonstrating data from the level of community hospitals from all districts nationwide, not limited to large centers with high research capacity.

Portal invasion of HCC is one of the most common causes of unresectability. Before sorafenib proved its efficacy in landmark randomized studies [6,7], possible modalities were limited to palliative or supportive care with expected survival of 2–4 months [19,20,21], which is comparable with the median survival of two months in the BSC group in our study. In a small Korean study that applied sorafenib monotherapy for 30 patients with portal invasion, the median OS was as mediocre as 3.1 months (95% CI 2.7–3.5) [22]. In a Japanese study comparing EBRT and sorafenib for HCC with main portal vein thrombosis using PS matching, EBRT demonstrated favorable survival outcomes (median period: 10.9 vs. 4.8 months; *p* = 0.025). In a recent meta-analysis, TACE for HCC patients with portal vein thrombosis showed a median OS period, pooled 1-year OS rate, response rate, and complication rate of eight months, 29% (95% CI: 20–40%), 19% (11–29%), and 18%, respectively [23].

EBRT has also been applied as an effective palliative modality. Compared with TACE, EBRT has the technical merit of delivering radiation to tumor volume regardless of location and vessel assessment; additionally, major vessels are tolerant to radiation, which can endure up to ~100 Gy [12]. Our team meta-analyzed the efficacy of radiotherapy modalities in palliating HCC with portal vein invasion [14]. Using three-dimensional conformal radiotherapy (3DCRT), which is the most common modality in recent decades, median OS, pooled 1-year OS, and response rate were 11.3 months, 43.8% (95% CI: 37.6–50.2) and 51.3% (45.7–57.0), respectively. With the application of stereotactic body radiotherapy (SBRT), which is an advanced modality with precise targeting, the correlating rates were 14.0 months, 48.5% (95% CI 39.4–57.8) and 70.7% (63.7–76.8) months, respectively. These survival results appeared to be more favorable than the correlating results of the present study (median and 1-year OS rates: eight months and 34.2%, respectively). The present study and the aforementioned meta-analysis both included a large number of patients with the same subject, and there did not appear to be distinct clinical profile differences between studies. Furthermore, both studies mostly included patients from the East Asian population. Therefore, we assumed that such differences in outcomes were caused by hospital factors; it is widely agreed in the literature that hospital and physician volume influence treatment outcomes of patients with cancer [24]. Several population-based studies on liver cancers reported that hospital volume was associated with improved survival, mediated by increased modality utilization or multidisciplinary evaluations, among others [25,26,27]. The meta-analysis recruited data from representative hospitals with abundant research capacity, whereas the present study included hospitals from all districts nationwide, hence representing the data from the community level.

Since the majority of patients had preserved liver function, we further categorized CPC A using the ALBI grade to evaluate differences in LRT benefits. LRT demonstrated benefits in both CPC-A1 and CPC-A2 patient groups, without significant differences in survival between the groups (*p* = 0.412), although the survival was numerically higher in the CPC-A1 group (1-year OS: 51.1% vs. 36.2% with LRT). These results necessitate further investigation of the prognostic differences between the CPC-A1 and CPC-A2 groups in a larger number of patients. However, the therapeutic benefit may be reduced due to the risk of hepatotoxicity in patients with impaired liver function such as those with CPC B [28]. The literature on clinical experience applying EBRT for CPC B cases is scarce; furthermore, most of them are SBRT series, targeting small tumors precisely, and sparing most of the remaining liver. In a Korean multicenter study (KROG1605), a total of 184 patients with CPC B including 66.3% of those with portal invasion underwent EBRT with a conventional fractionation scheme (not SBRT). The median OS and 1-year local control rates were 9.4 months and 58.9%, respectively; however, 43.5% of patients experienced radiation-induced liver disease [29]. The present study is significant in that it demonstrates a survival benefit of LRT for CPC B patients with portal invasion (HR: 0.46, 95% CI: 0.33–0.66, *p* < 0.001), using a PS-matched group as a control.

Limitations of the present study include the fact that the data did not encompass imaging studies or subtypes of portal vein invasion. The main portal vein invasion was not distinguished from portal branch invasion; hence, it did not allow for a more detailed analysis. Although we assumed that the majority of radiotherapeutic modalities were conventionally fractionated radiotherapy, a few cases of SBRT may have been included in the data and may not be distinguishable. However, as the majority of HCC cases with portal vein invasion have tumorous targets near the bowels, the overall prescribed dose might not significantly exceed that of fractionated radiotherapy. Furthermore, according to our team’s recent meta-analysis involving studies published until July 2017, the vast majority of radiotherapeutic modalities for HCC involving the portal vein were 3DCRT (22 studies involving 1903 patients, all from East Asian countries), whereas few (four studies involving 208 patients) were SBRT. Therefore, we can assume that the majority of radiotherapeutic modalities in the present study could be conventionally fractionated 3DCRT [14]. Since the registry data were recruited from as many as 50 hospitals, complication data were not acquired considering the possible subjectivity and motivation of participating researchers. Approximately two-thirds of patients in the LRT group received combined TACE; since subgroup analyses to evaluate the effect of TACE were marginally insignificant, the contribution as a local modality could not be ruled out.

## 5. Conclusions

In conclusion, LRT has significant benefits for OS and CSS in HCC patients with portal invasion. This is valid for patients with CPC A and B. The difference in the benefit of LRT to CPC A patients divided by ALBI class should be investigated in future studies by enrolling a larger number of patients. Our study is significant in that it represents community-level data from hospitals from all administrative districts in Korea, thereby demonstrating a comprehensible LRT benefit with the help of PS-matching comparison.

## Figures and Tables

**Table 1:** Patient characteristics according to propensity-score matching.

	Before Propensity-Score Matching			After Propensity-Score Matching		
Covariates	No Treatment (*n* = 932)	LRT (*n* = 231)	*p*-Value *	No Treatment (*n* = 222)	LRT (*n* = 222)	*p*-Value *
Age	60.4 ± 12.1	54.6 ± 10.4	<0.001	55.8 ± 11.1	54.4 ± 10.0	0.165
Sex			0.979			0.309
Male	802 (86.1)	198 (85.7)		199 (89.6)	191 (86.0)	
Female	130 (13.9)	33 (14.3)		23 (10.4)	31 (14.0)	
Etiology			<0.001			0.322
Other cause	317 (35.7)	50 (22.4)		60 (27.0)	50 (22.5)	
Hepatitis B virus	571 (64.3)	173 (77.6)		162 (73.0)	172 (77.5)	
Child-Pugh score	7.7 ± 1.9	6.1 ± 1.3	<0.001	6.1 ± 1.3	6.1 ± 1.3	0.944
Extrahepatic metastases			0.023			0.659
None	640 (68.7)	177 (76.6)		165 (74.3)	170 (76.6)	
Present	291 (31.3)	54 (23.4)		57 (25.7)	52 (23.4)	
Main tumor size			0.159			0.103
<10 cm	390 (42.0)	109 (47.4)		87 (39.2)	105 (47.3)	
≥10 cm	539 (58.0)	121 (52.6)		135 (60.8)	117 (52.7)	
Multiplicity			0.001			0.849
Multiple	565 (60.7)	111 (48.1)		104 (46.8)	107 (48.2)	
Single	366 (39.3)	120 (51.9)		118 (53.2)	115 (51.8)	
Alpha-fetoprotein, µg/mL			0.186			0.678
<400	349 (38.0)	99 (43.0)		88 (39.6)	94 (42.3)	
400~10,000	268 (29.2)	54 (23.5)		62 (27.9)	54 (24.3)	
≥10,000	301 (32.8)	77 (33.5)		72 (32.4)	74 (33.3)	

Variables are expressed as means ± standard deviations or *n* (%). LRT, local treatment including radiotherapy. * Chi-square test was performed.

**Table 2:** Predictors for mortality.

		Overall Survival				Cancer-Specific Survival			
		Univariate		Multivariate		Univariate		Multivariate	
Covariates	Rating	HR (95% CI)	*p* Value	HR (95% CI)	*p* Value	HR (95% CI)	*p*-Value	HR (95% CI)	*p* Value
LRT		0.46 (0.38–0.55)	<0.001	0.41 (0.32–0.52)	<0.001	0.47 (0.39–0.58)	<0.001	0.43 (0.34–0.54)	<0.001
Age	>60 years	1.78 (1.42–2.22)	<0.001	1.83 (1.40–2.39)	<0.001	1.78 (1.42–2.24)	<0.001	1.82 (1.40–2.38)	<0.001
Male		0.96 (0.71–1.28)	0.759			0.92 (0.68–1.25)	0.609		
Hepatitis B virus		1.11 (0.89–1.38)	0.376			1.12 (0.89–1.40)	0.331		
Extrahepatic metastases		1.87 (1.50–2.34)	<0.001	1.90 (1.42–2.53)	<0.001	1.90 (1.51–2.38)	<0.001	1.90 (1.42–2.54)	<0.001
Main tumor size	≥10 cm	1.51 (1.24–1.84)	<0.001	1.60 (1.26–2.05)	<0.001	1.51 (1.24–1.85)	<0.001	1.56 (1.22–2.00)	<0.001
Multiple tumors		1.23 (1.01–1.48)	0.038	1.00 (0.79–1.27)	0.980	1.27 (1.04–1.54)	0.018	0.98 (0.78–1.24)	0.882
Alpha-fetoprotein	µg/mL	1.17 (1.05–1.31)	0.006	1.22 (0.94–1.59)	0.073	1.17 (1.04–1.30)	0.008	1.24 (0.95–1.62)	0.072
Child-Pugh Class	A vs. B	1.47 (1.19–1.81)	<0.001	1.87 (1.40–2.50)	<0.001	1.43 (1.15–1.77)	0.001	1.78 (1.32–2.39)	<0.001
Performance status	≥2	1.89 (1.28–2.80)	0.001	1.01 (0.64–1.57)	0.981	1.87 (1.26–2.78)	0.002	1.00 (0.64–1.58)	0.992

HR, hazard ratio; CI, confidence interval; LRT, local treatment including radiotherapy; CPC, Child-Pugh class; ALBI, albumin-bilirubin.

## Data Availability

The data that support the findings of this study are available from the corresponding author, C.H.R., upon reasonable request.

## Supplementary Materials

The following are available online at https://www.mdpi.com/article/10.3390/biology10040326/s1, Table S1: Clinical characteristics of patients in the LRT only group and LRT with TACE group.
