# Supplementary material for: Benefits of Local Treatment Including External Radiotherapy for Hepatocellular Carcinoma with Portal Invasion
Source: Biology (Basel). 2021 Apr 14;10(4):326. doi: 10.3390/biology10040326 (PMC8070697; doi:10.3390/biology10040326)
Supplement: Supplementary file 1 [file biology-10-00326-s001.pdf]

Table S1. Clinical characteristics of patients in the LRT only group and LRT with TACE group.

**Covariates**	**LRT Only (*n* = 78)**	**LRT with TACE (*n* = 144)**	***p* Value**
Age	55.2 ± 11.3	53.9 ± 9.3	0.014
Gender			0.290
Male	64 (82.1)	127 (88.2)	
Female	14 (17.9)	17 (11.8)	
Etiology			0.324
Other cause	21 (26.9)	29 (20.1)	
Hepatitis B virus	57 (73.1)	115 (79.9)	
Child-Pugh score			
Extrahepatic metastases			0.083
None	54 (69.2)	116 (80.6)	
Present	24 (30.8)	28 (19.4)	
Main tumor size			0.216
<10cm	32 (41.0)	73 (50.7)	
≥10cm	46 (59.0)	71 (49.3)	
Multiplicity			0.168
Multiple	43 (55.1)	64 (44.4)	
Single	35 (44.9)	80 (55.6)	
Alpha-fetoprotein, ug/mL			0.193
<400	36 (46.2)	58 (40.3)	
400~10,000	22 (28.2)	32 (22.2)	
≥10,000	20 (25.6)	54 (37.5)	
Child-Pugh class			0.141
A	47 (60.3)	105 (72.9)	
B	30 (38.5)	37 (25.7)	
C	1 (1.3)	2 (1.4)	
Performance status			0.943
0 or 1	59 (95.2)	112 (94.9)	
2 or higher	3 (4.8)	6 (5.1)	

Variables are expressed as mean ± standard deviation or *n* (%). LRT, local treatment including radiotherapy; TACE, transarterial chemoembolization.
